# Spatial and Host-Related Variation in Prevalence and Population Density of Wheat Curl Mite (*Aceria tosichella*) Cryptic Genotypes in Agricultural Landscapes

**DOI:** 10.1371/journal.pone.0169874

**Published:** 2017-01-18

**Authors:** Anna Skoracka, Mariusz Lewandowski, Brian G. Rector, Wiktoria Szydło, Lechosław Kuczyński

**Affiliations:** 1 Population Ecology Lab, Institute of Environmental Biology, Faculty of Biology, Adam Mickiewicz University in Poznań, Poland; 2 Department of Applied Entomology, Faculty of Horticulture, Biotechnology and Landscape Architecture, Warsaw University of Life Sciences –SGGW, Warsaw, Poland; 3 Great Basin Rangelands Research Unit, USDA-ARS, Reno, Nevada, United States of America; Rutgers The State University of New Jersey, UNITED STATES

## Abstract

The wheat curl mite (WCM), *Aceria tosichella* Keifer, is a major pest of cereals worldwide that also comprises a complex of at least 16 genetic lineages with divergent physiological traits, including host associations and specificity. The goal of this study was to test the extent to which host-plant species and landscape spatial variation influence WCM presence and population density across the entire area of Poland (>311,000 km^2^). Three important findings arose from the results of the study. (1) The majority of WCM lineages analyzed exhibited variation in patterns of prevalence and/or population density on both spatial and host-associated scales. (2) Areas of occurrence and local abundance were delineated for specific WCM lineages and it was determined that the most pestiferous lineages are much less widespread than was expected, suggesting relatively recent introductions into Poland and the potential for further spread. (3) The 16 WCM lineages under study assorted within four discrete host assemblages, within which similar host preferences and host infestation patterns were detected. Of these four groups, one consists of lineages associated with cereals. In addition to improving basic ecological knowledge of a widespread arthropod herbivore, the results of this research identify high-risk areas for the presence of the most pestiferous WCM lineages in the study area (viz. the entirety of Poland). They also provide insight into the evolution of pest species of domesticated crops and facilitate testing of fundamental hypotheses about the ecological factors that shape this pest community.

## Introduction

All effective and sustainable management strategies rely on core ecological knowledge of the distribution and abundance of the target organism and the factors that influence these outcomes. Predicting species distributions and population densities has become a fundamental goal of many of basic and applied ecological disciplines [1–3], including practical applications such as conservation and wildlife management and forecasting parasite and pest population dynamics (e.g. [4–9]). Niche models that are routinely employed for predicting species distributions often focus solely on abiotic factors when evaluating the potential habitat suitability for a species of interest (e.g. [10, 11]). However, consideration of biotic interactions in modelling species distributions should improve our ability to predict distribution patterns [12]. This is especially important for organisms (i.e. parasites or plant pests) that have co-evolved under facultative or obligatory associations with other organisms (i.e. hosts) and whose distribution and abundance depend intrinsically on those of their hosts. As such, a central question regarding spatial variation of host-associated species concerns the extent to which their distribution and population density are determined by host-related variation or by geographic variation. In general, the interplay of spatial- and host-associated variation on species distribution and abundance has not been adequately addressed (e.g. [13]). In the present study, we explore this question using the herbivorous mite *Aceria tosichella* Keifer (wheat curl mite; WCM hereafter) as a study system. This mite occurs in nature in a variety of genetically divergent lineages (i.e. genotypes; likely cryptic species) that are associated with numerous grass hosts in both host-specific and generalist relationships [14–16].

Despite the great economic importance of WCM as a worldwide pest of cereals [17, 18] its large-scale distribution and abundance have not been studied. This shortcoming hinders WCM management strategies by reducing our ability to predict herbivore pressure. The major negative impact of WCM is its ability to vector several economically important plant viruses, including *wheat streak mosaic virus* (WSMV), a major global pathogen of wheat for which WCM is the only known arthropod vector. This potyvirus may cause as much as 100% yield loss in wheat; it can also reduce yields in barley, oats and rye [18, 19]. The spread of this WCM-virus nexus to cereal producing regions worldwide is of increasing importance, as it continues to impact regions invaded long ago (e.g. North America), while also emerging as a pest in Australia, South America and Europe (e.g. [18, 20–24]). Thus, a comprehensive understanding of WCM distribution and abundance dynamics is of great importance to cereal pest managers as well as ecologists, as information gathered from such systems is essential to advance basic knowledge of species occurrence and population density.

The current lack of core ecological data for the WCM complex is due in part to the fact that detection and identification of WCM genotypes in environmental samples is technically challenging. The mites are tiny (ca. 0.2 mm) and the various genotypes exhibit intraspecific morphological homogeneity. Therefore, their identification is currently based on DNA barcoding using the mitochondrial COI marker gene as well as nuclear regions, e.g. 28S rDNA D2 [25, 26]. Since the cryptic diversity of WCM is a relatively recent discovery [14–16], the majority of ecological data to date have been recorded for WCM sensu lato, rather than for individual genotypes. Such lack of precision may have serious consequences, as genotypes within species complexes may differ with respect to host range, life-history traits, tolerance to environmental factors, pesticide resistance, ability to transmit diseases and other key biological traits (e.g. [27, 28]). The host specificity of some WCM lineages has been experimentally tested and divergent host preferences, ranging from specialists to generalists, have been observed [29]. Moreover, the most polyphagous WCM genotypes currently known (designated as MT-1 and MT-8) are distributed globally and considered to have the greatest pest and invasion potential [25, 30, 31].

In this study the host and spatial patterns of WCM genotype prevalence and abundance was presented for the first time on a broad landscape scale (>300 000 km^2^), on the basis of extensive spatially-explicit sampling of quantitative data and DNA barcoding. Specifically, we described how WCM genetic lineages in Poland vary in space and with respect to host plant. First, considering the obligatory associations of WCM lineages with their host plants we hypothesized that (i) the host plant is the main factor influencing prevalence and population density of non-monophagous WCM genotypes, i.e. that these measures will reflect a relative preference between different host species, whereas (ii) landscape spatial variation should be the primary factor affecting prevalence and population density of monophagous WCM genotypes. Second, we expected that two globally spread WCM genotypes (viz. MT-1 and MT-8), due to their pest and invasion potential and acceptance of multiple grass species as hosts [25], are widely distributed and abundant across the whole area of Poland. This assumption is additionally supported by previous studies showing that the majority of Poland offers optimal thermal conditions for the development of both lineages [32]. Third, we hypothesized that genetic lineages within the WCM complex avoid competition through the partitioning of resources in terms of host species or spatial distribution. We expected that, according to the competitive exclusion principle [33], parameters of infestation of particular WCM genotypes will reflect different host preferences or different patterns of prevalence and abundance in the studied area.

## Methods

### Sampling design

The study did not include human participants and/or tissue, animals, embryos or tissues. The study did not require field permit. The field survey was carried out on the entire area of Poland, which covers > 311,000 km^2^, from June to August of 2013 & 2014. The localities accessed were not protected. To achieve an even distribution of sampling localities, a stratified random sampling scheme was employed. The studied area was divided into squares, each measuring 30 x 30 km, to form spatial strata. Within each stratum a 1 x 1 km square of agrarian landscape was selected at random. Randomisation was restricted to agrarian cover types based on the Corine Landcover database [34]. Altogether, 362 squares were sampled. For map and other details see [32]. Names of the sampling locations and their geographic coordinates are held in the public repository Zenodo under DOI:10.5281/zenodo.168045.

Eight species of cereals and unprotected wild grasses commonly associated with agrarian landscapes and evenly distributed within Poland [35] were collected from the centre of each 1 km^2^ square. These were: wheat (*Triticum aestivum* L.), triticale (*xTriticosecale* Wittm), rye (*Secale cereale* L.), oat (*Avena sativa* L.), barley (*Hordeum vulgare* L.), quackgrass (*Elymus repens* (L.) Gould), tall oat-grass (*Arrhenatherum elatius* (L.) Beauv. ex Presl & Presl), and smooth brome (*Bromus inermis* Leyss). Each sample consisted of at least ten plant shoots of a given grass species. Shoots were placed separately in plastic bags, to protect from drying, labelled and transported to the laboratory (in coolers when necessary). A total of 1253 samples were collected, which comprised 10,965 grass shoots. Samples were stored at 4°C ± 1°C for a maximum of five days before being examined for the presence of mites under a stereo-microscope. Entire plants were inspected and the number of WCM specimens found was recorded, after which WCM specimens were soaked in ATL buffer for subsequent DNA identification.

### DNA barcoding

DNA was extracted from mite specimens that had been stored in ATL buffer according to the non-destructive method described in [36]. The mitochondrial *cytochrome c oxidase subunit I* (CO1) gene fragment was amplified by PCR using the degenerate primers bcdF01 and bcdR04 [37]. Reactions were carried out in 10 μL reaction volumes containing 5 μL Type-it Multiplex PCR Master Mix (Qiagen GmbH), 0.5 μL of each primer and 4 μL of DNA template. Reactions were initially heated for 5 min at 95°C followed by 35 cycles of 30 s at 95°C, 30 s at 50°C, and 1 min at 72°C; with a final step of 15 min at 72°C. Amplicons were diluted (10 μL dH2O: 3 μL amplicon) and analysed by electrophoresis in 1% agarose gels. Samples presenting visible, individual bands were directly sequenced in both directions using 0.5–1 μL of amplicon and 50 pmol of each primer. Sequencing was performed with BigDye Terminator v3.1 on an ABI Prism 3130XL or 3730 Analyzer (Applied Biosystems, Foster City, CA, USA). Trace files were checked and edited using MEGA6 [38]. A subsequent BLAST search of each sequence was performed on the NCBI GenBank database. Sequences of WCM lineages were aligned using CLUSTALW in MEGA6 with default gap weighting parameters. Alignment of CO1 sequences covering 603 bp of the 5-terminus of the COI gene fragment was checked by translating aligned DNA sequences into amino acids. Neighbour-joining analysis was performed in MEGA6 to visualize the clustering of novel haplotypes (i.e. lineages) (data not shown). Uncorrected p-distances with standard error estimates (obtained using a bootstrap procedure with 1000 replicates) were calculated between and within WCM clusters using MEGA6 and new lineages were declared when inter-cluster divergence was at least 10x the intra-cluster variation [39]. Sequences have been deposited in NCBI GenBank under the accession numbers KX430258 to KX430320. Following DNA extraction, mite exoskeletons were preserved in 70% ethyl alcohol. In cases where obtained sequences did not match any previously recorded WCM lineages [25, 26], exoskeletons were mounted on slides and morphologically identified using a phase-contrast microscope.

### Statistical analysis

To test how the prevalence (i.e. probability of occurrence) and population density (i.e. the number of individuals per shoot) of different WCM genetic lineages was related to host species and how they varied spatially, a generalized additive model (GAM) was used [40]. This method is well suited to test our hypothesis, as it can incorporate in one model both categorical predictors (host species) and spatial smoothing (geographic location). Splines used in GAM allow to flexibly fit complicated, nonlinear functions and thus are well suited to model spatial patterns.

The experimental unit in the analysis was a single shoot. For each WCM genetic lineage two separate models were fitted: one for prevalence (with Bernoulli distribution for the response and the logit link function) and one for population density (with Tweedie distribution for WCM counts and the log link function). Predictors were: host species (categorical, parametric predictor) and geodetic coordinates (smooth two-dimensional splines). Additionally, to account for uneven sampling effort, the natural log—of the number of leaves in each sample was used as an offset.

Maps were produced by predicting mite presence and abundance for the most abundant host at a given site based on the GAM models. The values on maps represent the predicted probability of occurrence or population log-density per shoot. Host-niche similarities between WCM lineages were expressed as Pearson correlation coefficients between population densities calculated for each WCM lineage on the various host species.

Statistical analysis was performed in R version 3.3 [39] using the mgcv 1.8 package [41]. Heat-maps showing similarity matrices were produced with the pheatmap package [42].

## Results

### Host infestation and host niche similarity of WCM genotypes

Overall, 16 WCM lineages were collected across Poland, including seven identified in the course of previous studies (MT-1 to MT-6 and MT-8) [25] and nine new lineages (designated as MT-9, MT-10, MT-12 to MT-15, and MT-27 to MT-29). The uncorrected p-distances between lineages ranged from 6.7% to 30.4%, whereas the intra-lineage diversity ranged from 0 to 2.4% (S1 Fig). All sampled plant species, with the exception of rye, hosted more than one WCM lineage although they differed in infestation parameters. Nine WCM lineages were associated with only one host plant species (hereafter ‘monophagous’). Four genotypes (MT-2, MT-4, MT-12, MT-13) were each found on two hosts, although MT-2 and MT-4 were found predominantly on quackgrass and only once each on wheat and were thus considered quackgrass-associated monophagous lineages, whereas MT-12 and MT-13 were considered as ‘oligophagous’. Three genotypes (MT-1, MT-3, MT-8) were collected from several hosts (‘polyphagous’). Among monophagous lineages, MT-9, MT-10 and MT-14, all were associated with smooth brome, with MT-9 attaining the highest population density. The highest infestation parameters on quackgrass were reached by the polyphagous MT-3, and on tall oat-grass by the monophagous MT-5. Cereals were predominantly occupied by MT-1 and MT-8 (S2 Fig; S1 Table).

**Fig 1:**
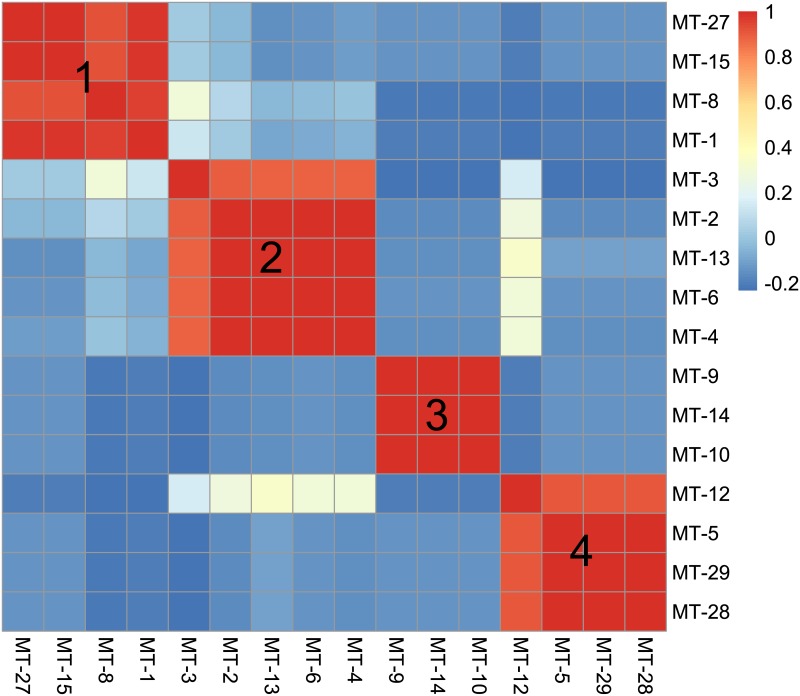
The heatmap representing host niche similarity measured as correlation between population log-densities of particular wheat curl mite (WCM) lineages on grass host species. Correlation coefficient is expressed with colors explained on the scale on the left. Numbers indicate the groups of WCM lineages that clustered according to host associations (1- with cereals, 2 –with quackgrass, 3 –with smooth brome, 4 –with tall oat-grass).

The WCM lineages clustered into four groups by host preference (Fig 1). Group 1 included lineages associated with cereals, with two polyphagous genotypes predominant on cereals and two monophagous lineages found only on wheat. Groups 2 and 3 comprised lineages associated with quackgrass and smooth brome, respectively. The fourth group was represented by tall-oat-grass-associated specialists and the oligophagous MT-12. There was no correlation between host niches for lineages MT-3 and MT-8, nor between MT-12 and any genotype belonging to Group 2. Additionally, the lineage MT-12 showed a strong negative correlation with genotypes belonging to Groups 1 and 3. Finally, the three polyphagous lineages (MT-1, MT-3 and MT-8) negatively correlated with the smooth brome- and tall-oat-grass-associated groups (3 and 4) (Fig 1).

### The effect of host and spatial variation on infestation parameters of WCM genotypes

**Fig 2:**
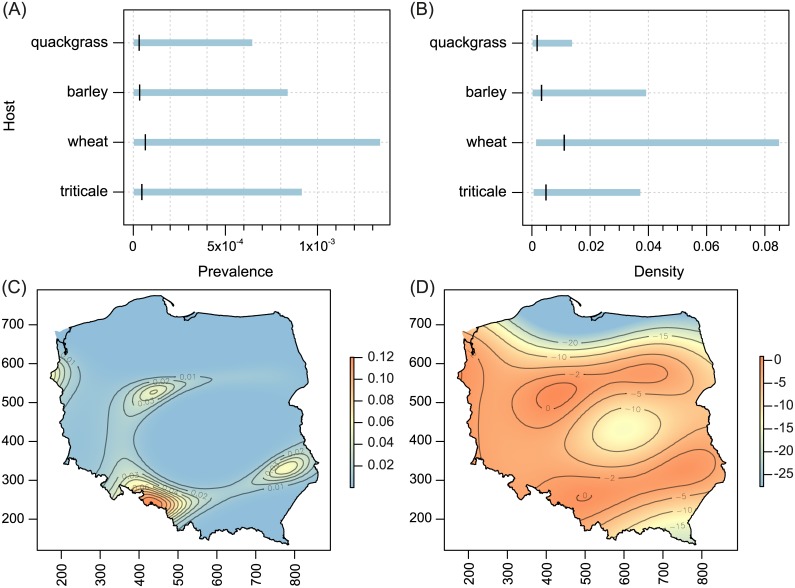
Host-related differences in prevalence (A) and population density (B), and spatial patterns of prevalence (C) and population density (D) for WCM MT-1 lineage. On (A) and (B), the black vertical tick represents the mean, and the blue horizontal bars are 95% confidence intervals. On (C) and (D), x-axis: increasing values more easterly, y-axis: increasing values more northerly. Colors on the maps represent fitted trend surfaces. For the prevalence they are expressed on the predictor scale (so they are predicted probabilities of occurrence) and for the density (i.e. the number of individuals per shoot) they are on the linear predictor scale (showing the logarithms of the predicted population densities).

**Fig 3:**
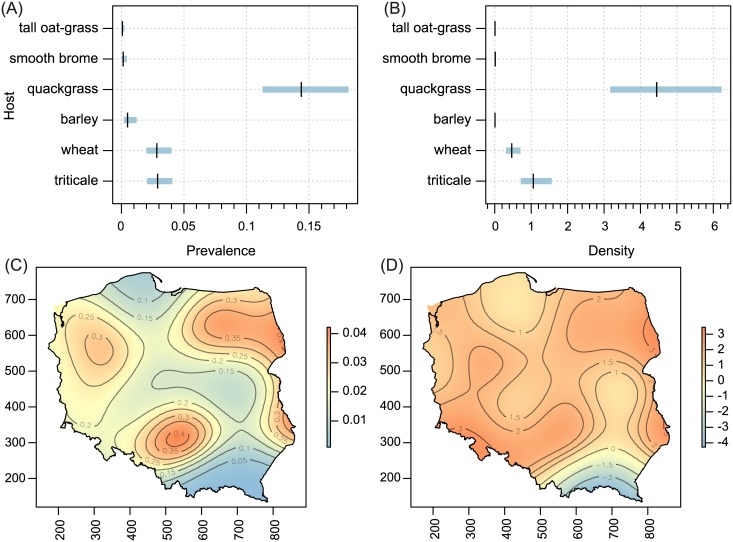
Host-related differences in prevalence (A) and population density (B), and spatial patterns of prevalence (C) and population density (D) for WCM MT-3 lineage. For explanation see Fig 2.

**Fig 4:**
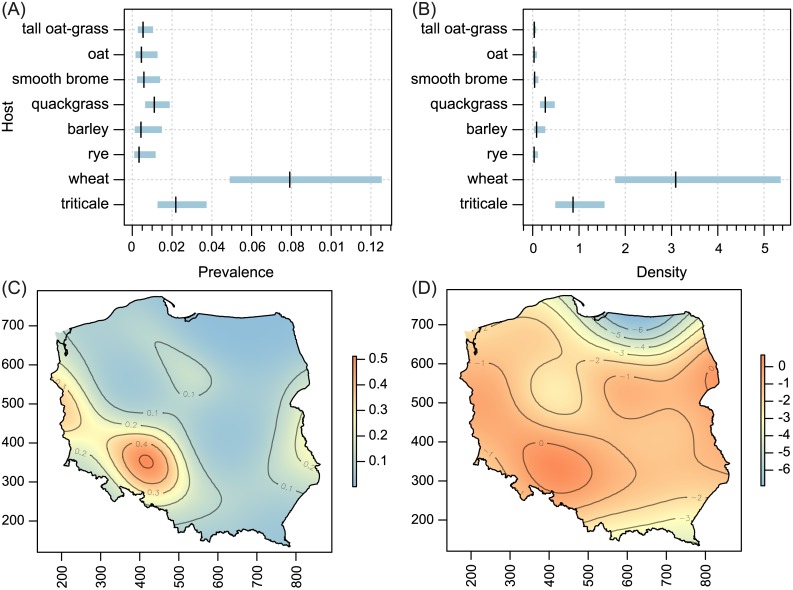
Host-related differences in prevalence (A) and population density (B), and spatial patterns of prevalence (C) and population density (D) for WCM MT-8 lineage. For explanation see Fig 2.

**Fig 5:**
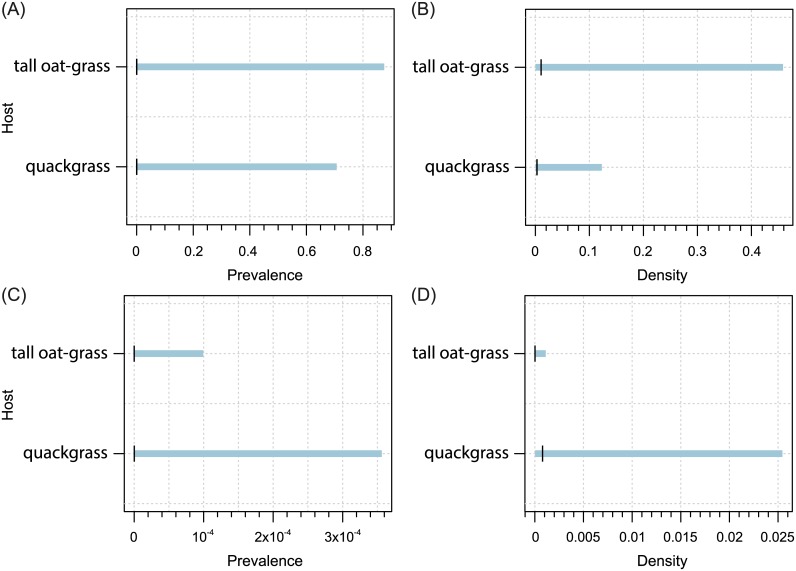
Host-related differences in prevalence (A, C) and population density (B, D) for WCM MT-12 (A, B) and MT-13 (C, D) lineages. For explanation see Fig 2.

**Fig 6:**
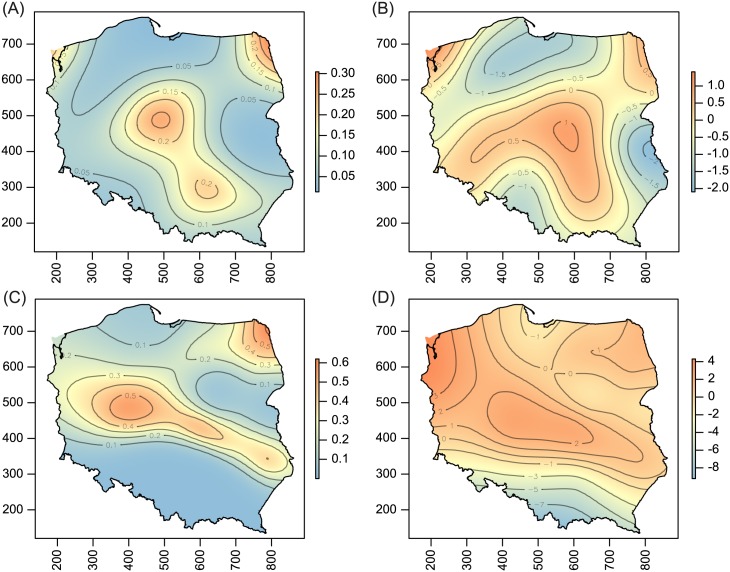
Spatial patterns of prevalence (A, C) population density (B, D) for WCM MT-5 (A, B) and MT-9 (C, D) lineages. For explanation see Fig 2.

**Fig 7:**
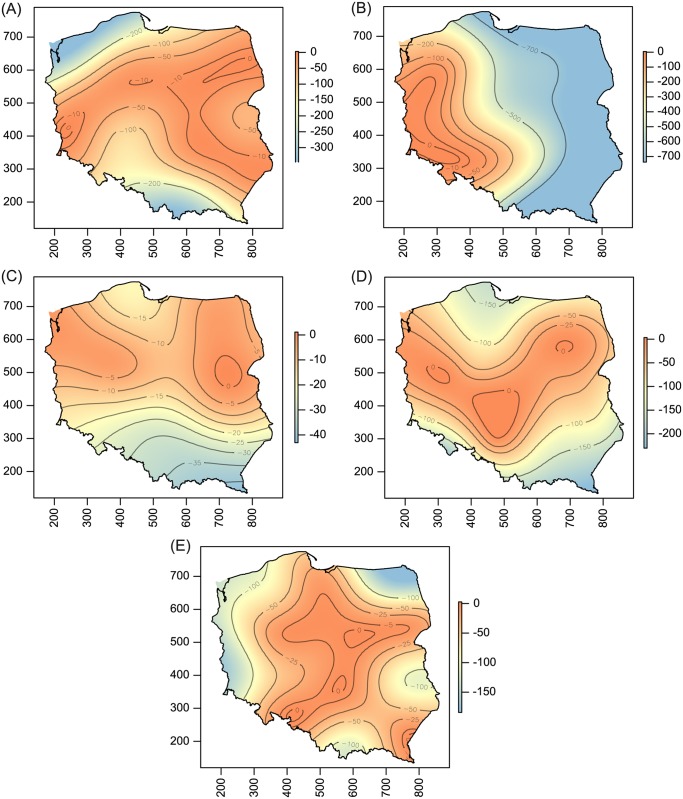
Spatial patterns of population density for WCM MT-2 (A), MT-4 (B), MT-6 (C), MT-10 (D) and MT-13 (E) lineages. For explanation see Fig 2.

Host-plant species significantly influenced both prevalence and population density of all polyphagous WCM lineages and the oligophagous lineage MT-13, as well as population density of MT—12 (Tables 1 and 2; Figs 2–5). The prevalence of MT-12 was not significantly different between its two hosts (tall oat-grass and quackgrass). There was significant spatial variation in prevalence in eight out of 13 genetic lineages tested (Table 1). Population density also showed a substantial amount of spatial variation; the trend surface was a significant predictor in 10 of 13 lineages (Table 2). Maps of probability of occurrence (prevalence) and predicted population densities are presented for all WCM lineages having a significant effect of spatial variation on infestation parameters (Figs 2–4, 6 and 7).

**Table 1:** Parameters of the generalized additive model examining the prevalence of genetic WCM lineages (expressed as proportion of infested shoots) in relation to the host plant species and geographic coordinates (which represent spatial variation).

	Host plant			Trend surface (spatial pattern)		
Lineage	df	Chi-sq.	p	edf	F	p
MT-1	4	74.34	<0.0001	18.8	83.8	<0.0001
MT-2				18.7	17.1	0.5820
MT-3	6	6918.0	<0.0001	18.4	200.0	<0.0001
MT-4				13.9	24.0	0.0494
MT-5				16.0	64.3	<0.0001
MT-6				18.8	31.8	0.0348
MT-8	8	3140.0	<0.0001	18.2	261.4	<0.0001
MT-9				17.6	59.8	<0.0001
MT-10				10.6	3.8	0.9750
MT-12	2	4.4	0.1750	15.3	21.9	0.1750
MT-13	2	14.7	0.0006	18.8	35.2	0.0132
MT-14				5.9	2.6	0.8530
MT-27				4.4	2.3	0.7900

Estimated degrees of freedom, “edf”, reflect the smoothness of the fitted surface. Degrees of freedom for the host plant are equal to the number of hosts that were found to infest a given lineage. If there was only one host found or two hosts among which one had only single shoot infested, this value cannot be determined, as there is no host—associated variability.

**Table 2:** Parameters of the generalized additive model examining the population density of WCM genetic lineages (expressed as number of specimens per shoot) in relation to the host plant species and geographic coordinates (which represent spatial variation).

	Host			Trend surface (spatial pattern)		
Lineage	df	Chi-sq.	p	edf	F	p
MT-1	4	34.08	<0.0001	18.1	6.16	<0.0001
MT-2				17.3	3.70	<0.0001
MT-3	6	185.6	<0.0001	18.3	17.30	<0.0001
MT-4				10.2	5.55	<0.0001
MT-5				14.4	2.42	0.0010
MT-6				12.7	3.29	<0.0001
MT-8	8	123.5	<0.0001	17.4	9.75	<0.0001
MT-9				16.5	8.97	<0.0001
MT-10				11.5	2.54	0.0027
MT-12	2	7.4	0.0006	12.6	1.52	0.0994
MT-13	2	14.4	<0.0001	17.6	2.54	0.0002
MT-14				6.7	1.16	0.3240
MT-27				6.6	0.00	1.0000

See Table 1 for explanations.

## Discussion

The general paucity of basic ecological studies is one of the main gaps in existing knowledge of the wheat curl mite (WCM) complex, hindering its monitoring and the prediction of its occurrence. The results of this study reveal the ecological complexity of a herbivorous mite species, *A*. *tosichella* sensu lato (WCM), that appears to be in the midst of a species radiation event in which distinct genotypes appear to be separated pre-zygotically due to divergent host preferences [15, 29]. This complexity was also found to extend to spatial distributions of the diverging WCM genotypes over the area of Poland (> 300,000 km^2^), independent of the distributions of their hosts, which were each widespread across the study area. Extensive sampling and a comprehensive dataset reveal for the first time fundamental information on the distribution and abundance of currently known WCM genotypes in European agricultural landscapes. Three important findings emerged from this descriptive study.

First, we found that in addition to genetic differences and divergent host preferences (e.g. [29]) the majority of WCM lineages exhibit variation in patterns of infestation parameters on both spatial and host scales. The obligatory dependence of phytophagous organisms on plants suggests that host plant availability should be among the most important factors affecting spatial distribution of herbivore populations [12, 43]. In this study WCM infestation parameters were tested for plant species that were both common and widespread across the study area, allowing a comparison of the effects of host versus geographic variation. Contrary to our expectation, the results showed that the host plant was the main factor influencing population density of only one oligophagous WCM genotypes, namely MT-12. Whereas, population density and prevalence of three polyphagous WCM lineages and oligophagous MT-13 was mediated by both host species and spatially (i.e. geographically) determined factors. Therefore, a deeper understanding of biotic and abiotic characteristics (e.g. microclimate, topography, environmental heterogeneity, natural enemies, competitors) that may influence spatial geographic variation of these WCM lineages will be necessary to elucidate the mechanisms underlying their population dynamics. However, geographic location alone can also have a dominant effect on distribution of organisms, as was shown for bees, spiders, earthworms and plants in European agricultural grasslands [44]. The population density of six and abundance of four among eight monophagous lineages were affected by spatial variation (Tables 1and 2), which partially supported our predictions. Neither the abundance nor prevalence of the MT-14 or MT-27 lineages, nor the prevalence of the MT-2 or MT-10 lineages were correlated with geographical variation suggesting that they were accidental, with no spatial factors affecting infestation parameters. Instead, infestation parameters of these lineages may be influenced by other factors, such as e.g. biotic interactions on a smaller scale. The outcomes of this research facilitate further studies on distribution and population dynamics of WCM genotypes to determine the scale and type of data (e.g. geographically-related vs. trophic interplay) that need to be considered.

The second important result of this study was the recognition of areas of occurrence and varied abundance for particular WCM lineages on an extensive landscape scale (viz. the entirety of Poland, the eighth largest country in Europe). From an applied point of view, spatial distribution maps are especially important for lineages associated with cereals (Group 1, Fig 1), and among them particularly for the two polyphagous and most pestiferous MT-1 and MT-8 [25]. Contrary to our expectations of their high frequency and wide distribution across the country, results showed only four areas of high prevalence, suggesting that these genotypes may have been introduced into Poland recently. Interestingly, the pattern of their prevalence appears to reflect the path of their dispersal in Poland though a pass between the Sudeten and Carpathian ranges (the Moravian Gate), in addition to the western part of the country (Figs 2C and 4C), which are the warmest regions in Poland [45]. Indeed, the population density patterns of MT-1, MT-8 and additionally MT-3 (which also sporadically infest cereals) suggest that these lineages fare poorly in cold regions (see Figs 2D, 3D and 4D), including the north central (Gulf of Gdańsk) and northeastern regions and southern mountain ranges. This pattern is consistent with a recent study that modeled the most thermally suitable areas in Poland for population development of MT-1 and MT-8, which showed that the majority of Poland offers optimal thermal conditions for growth of both lineages from May to September [32].

Integrating a spatial component into ecological research on the WCM complex, combined with the identification of high-risk areas for pestiferous lineages [32] should enable prediction of invasions and development of WCM management strategies at landscape scales. Knowledge of the distribution and greatest abundance of lineages MT-1 and MT-8 is crucial for prediction of future outbreaks of these pests. With predicted rise in global temperatures, population densities of these lineages should be monitored, as their polyphagy may enhance their ability to occupy new regions, as was shown for a polyphagous butterfly in Britain [46]. Considering the low values of infestation parameters of two other cereal-associated lineages, MT-15 and MT-27 (S1 Table), it seems likely that they were accidentally present on wheat, a possible artifact of collection bias within agrarian landscapes. In addition, the existence of monophagous specialists on cereal species would be quite unexpected, given the recent creation of these grass species by human hands. It is likely that these lineages occupy hosts that were not sampled in this study.

The third notable outcome arising from the results of this study is the discovery of four host-assemblages among the 16 studied WCM lineages, within which similar host preferences and host infestation patterns were detected. The association of several (from three to five) genetically distinct (COI p-distance >11%) WCM lineages sharing the same host species (Fig 1, S1 Fig) was unexpected. This raises the question of connectivity between different WCM genotypes and the mechanisms of their genetic divergence and speciation. In the case of WCM genotypes associated with different plant species, ecological isolation via host-associated divergence can function as an effective barrier to gene flow (e.g. [47–49]). Conversely, different WCM genotypes sharing the same host species are more likely to make contact, so gene flow between them should be possible. However, even if gene flow between WCM genotypes is ongoing, post-zygotic barriers may act by reducing hybrid fitness, thus driving the evolution of pre-zygotic isolation [50]. This phenomenon was observed in two cryptic populations of another eriophyoid species complex [51]. The extent of gene flow between WCM lineages has not been tested but such studies are warranted for WCM lineages that share both host and spatial distribution, including MT-1 and MT-8 on wheat, MT-9 and MT-10 on smooth brome, and all quackgrass-associated lineages (Group 2, Fig 1).

Sympatry of genotypes or cryptic species within a species complex, such as in WCM, may be explained by occupation of different niches on the host-plant (allotropy sensu [52]) or allopatric speciation, i.e. reuniting after a long period of geographic isolation (e.g. [53]). Alternatively, a new species that evolves by occupying a new host could be brought back into contact with its progenitor species following an identical host shift by the latter [54]. If reproductive incompatibility between the two genotypes remains incomplete, gene flow may recommence, possibly followed by speciation reversal (sensu [55]). If reproductive incompatibility is complete, the two cryptic species will simply co-occur.

According to the competitive exclusion principle, interacting species within a community require sufficient genetic, phenotypic and ecological disparity to coexist [33]. Thus, depending on the extent of the ecological similarity between WCM cryptic genotypes, such sympatry may persist or one may displace the other. Partitioning resources within a host (i.e. multiple niches on a single host) could allow WCM genotypes to minimize competition and consequently coexist. Moreover, competitive interactions may affect geographic ranges especially between closely related species [12]. This may explain why distributions of WCM lineages associated with the same host species, while overlapping, are not in fact identical.

In addition to improving basic ecological knowledge of a widespread arthropod herbivore, the results of this research identify high-risk areas for the presence of the most pestiferous WCM lineages in the study area (viz. the area of Poland). The results raise many important questions for future laboratory and field investigations, e.g. regarding abiotic and biotic factors affecting the distributions of particular WCM genotypes, competitive displacement and coexistence between closely related genotypes, the role of host plant in gene flow between genotypes (including domesticated vs. wild host species), the possible range expansion of the pestiferous WCM lineages, and the factors, both ecological and genetic, that lead to the development of pest species of domesticated crops.

## Supporting Information

S1 Fig Heat map representing pairwise uncorrected p—distances for mtDNA COI gene between all currently detected wheat curl mite (WCM) lineages in Poland and *Trisetacus silvestris* (GenBank Acc nos. KC776549-KC776550) as an outgroup species. Up to five sequences representing each WCM lineage were used (GenBank Acc nos: KX430258 to KX430320).
(PDF)

S2 Fig Density (with 95% confidence intervals) of wheat curl mite (WCM) lineages on the studied host plant species. (PDF)

S1 Table Infestation parameters of the wheat curl mite (WCM) genetic lineages on different host plants. P—prevalence [percentage of shoots infested], I—intensity [mean number of specimens per infested shoot], D—density [mean number of specimens per all shoots] with 95% confidence intervals [CI], n—number of all shoots under study, k—number of shoots infested by mites.
(PDF)
