# Supplementary figures and images for: Spatial and Host-Related Variation in Prevalence and Population Density of Wheat Curl Mite (Aceria tosichella) Cryptic Genotypes in Agricultural Landscapes
Source: PLoS One. 2017 Jan 18;12(1):e0169874. doi: 10.1371/journal.pone.0169874 (PMC5242520; doi:10.1371/journal.pone.0169874)

S1 Fig

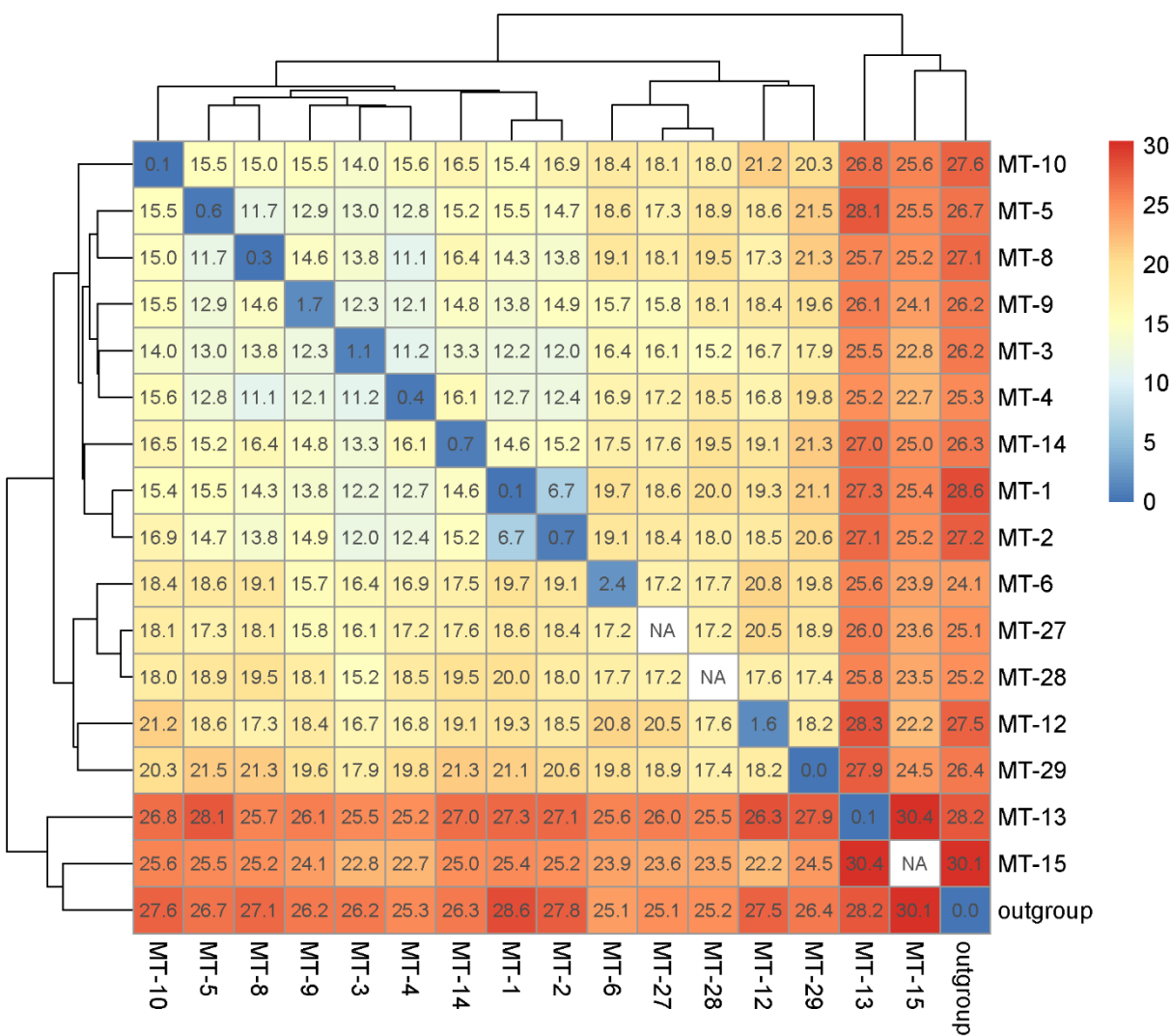

Supplement: S1 Fig — Up to five sequences representing each WCM lineage were used (GenBank Acc nos: KX430258 to KX430320). (PDF) [file pone.0169874.s001.pdf]

**S2 Fig**

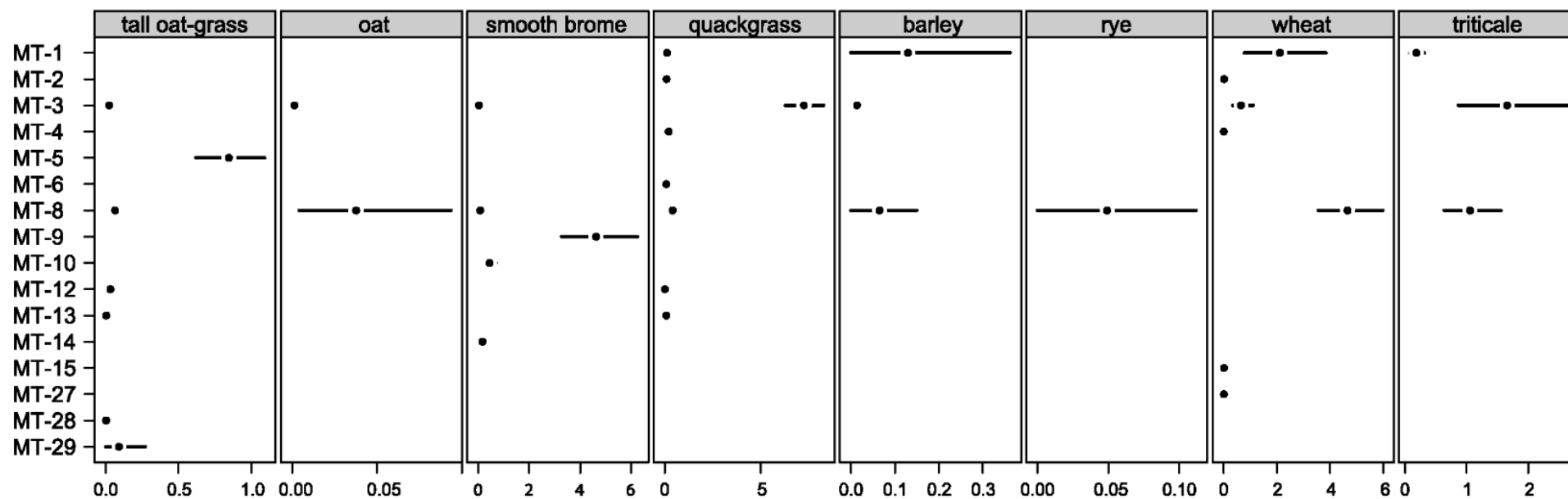

Supplement: S2 Fig — (PDF) [file pone.0169874.s002.pdf]
