# Supplementary material for: Spatial and Host-Related Variation in Prevalence and Population Density of Wheat Curl Mite (Aceria tosichella) Cryptic Genotypes in Agricultural Landscapes
Source: PLoS One. 2017 Jan 18;12(1):e0169874. doi: 10.1371/journal.pone.0169874 (PMC5242520; doi:10.1371/journal.pone.0169874)
Supplement: S1 Table — P—prevalence [percentage of shoots infested], I—intensity [mean number of specimens per infested shoot], D—density [mean number of specimens per all shoots] with 95% confidence intervals [CI], n—number of all shoots under study, k—number of shoots infested by mites. (PDF) [file pone.0169874.s003.pdf]

S1 Table

WCM Lineage	Host plant species	n	k	P	95% CI	I	95% CI	D	95% CI
MT-1	wheat	1641	38	2.32	1.66–3.12	91.29	37.71–154.92	2.11	0.78–3.85
	quackgrass	3659	39	1.07	0.77–1.43	11.15	6.56–17.67	0.12	0.06–0.20
	triticale	1531	15	0.98	0.57–1.56	18.87	11.73–26.40	0.18	0.08–0.31
	barley	447	3	0.67	0.17–1.73	19.33	4.00–43.00	0.13	0.00–0.36
	rye	509	0	0.00	0.00–0.66	0.00	0.00–0.00	0.00	0.00–0.00
	smooth brome	651	0	0.00	0.00–0.52	0.00	0.00–0.00	0.00	0.00–0.00
	oats	716	0	0.00	0.00–0.47	0.00	0.00–0.00	0.00	0.00–0.00
	tall oat-grass	1811	0	0.00	0.00–0.19	0.00	0.00–0.00	0.00	0.00–0.00
MT-2	quackgrass	3659	32	0.87	0.61–1.21	11.72	6.28–19.78	0.10	0.05–0.18
	wheat	1641	1	0.06	0.00–0.27	17.00	0.00–0.00	0.01	0.00–0.03
	barley	447	0	0.00	0.00–0.75	0.00	0.00–0.00	0.00	0.00–0.00
	rye	509	0	0.00	0.00–0.66	0.00	0.00–0.00	0.00	0.00–0.00
	smooth brome	651	0	0.00	0.00–0.52	0.00	0.00–0.00	0.00	0.00–0.00
	oats	716	0	0.00	0.00–0.47	0.00	0.00–0.00	0.00	0.00–0.00
	triticale	1531	0	0.00	0.00–0.22	0.00	0.00–0.00	0.00	0.00–0.00
	tall oat-grass	1811	0	0.00	0.00–0.19	0.00	0.00–0.00	0.00	0.00–0.00
MT-3	quackgrass	3659	900	24.60	23.22–26.01	29.45	25.92–33.14	7.24	6.28–8.27
	triticale	1531	73	4.77	3.78–5.91	34.82	19.74–53.82	1.66	0.87–2.66
	wheat	1641	74	4.51	3.58–5.59	14.42	8.16–24.09	0.65	0.34–1.11
	barley	447	5	1.12	0.40–2.39	1.20	1.00–1.60	0.01	0.00–0.03
	smooth brome	651	3	0.46	0.12–1.19	4.67	1.00–7.00	0.02	0.00–0.05
	tall oat-grass	1811	3	0.17	0.04–0.43	14.33	12.00–16.00	0.02	0.00–0.06
	oats	716	1	0.14	0.01–0.61	1.00	0.00–0.00	0.00	0.00–0.00
	rye	509	0	0.00	0.00–0.66	0.00	0.00–0.00	0.00	0.00–0.00
MT-4	quackgrass	3659	35	0.96	0.67–1.31	21.89	14.2–30.83	0.21	0.11–0.33
	wheat	1641	1	0.06	0.00–0.27	8.00	0.00–0.00	0.00	0.00–0.01
	barley	447	0	0.00	0.00–0.75	0.00	0.00–0.00	0.00	0.00–0.00
	rye	509	0	0.00	0.00–0.66	0.00	0.00–0.00	0.00	0.00–0.00
	smooth brome	651	0	0.00	0.00–0.52	0.00	0.00–0.00	0.00	0.00–0.00
	oats	716	0	0.00	0.00–0.47	0.00	0.00–0.00	0.00	0.00–0.00
	triticale	1531	0	0.00	0.00–0.22	0.00	0.00–0.00	0.00	0.00–0.00
	tall oat-grass	1811	0	0.00	0.00–0.19	0.00	0.00–0.00	0.00	0.00–0.00
MT-5	tall oat-grass	1811	146	8.06	6.87–9.38	10.49	8.2–13.08	0.85	0.62–1.09
	barley	447	0	0.00	0.00–0.75	0.00	0.00–0.00	0.00	0.00–0.00
	rye	509	0	0.00	0.00–0.66	0.00	0.00–0.00	0.00	0.00–0.00
	smooth brome	651	0	0.00	0.00–0.52	0.00	0.00–0.00	0.00	0.00–0.00
	oats	716	0	0.00	0.00–0.47	0.00	0.00–0.00	0.00	0.00–0.00
	triticale	1531	0	0.00	0.00–0.22	0.00	0.00–0.00	0.00	0.00–0.00
	wheat	1641	0	0.00	0.00–0.21	0.00	0.00–0.00	0.00	0.00–0.00
	quackgrass	3659	0	0.00	0.00–0.09	0.00	0.00–0.00	0.00	0.00–0.00

S1 Table continued

WCM Lineage	Host plant species	n	k	P	95% CI	I	95% CI	D	95% CI
MT-6	quackgrass	3659	25	0.68	0.45–0.99	11.84	7.72–16.52	0.08	0.04–0.13
	barley	447	0	0.00	0.00–0.75	0.00	0.00–0.00	0.00	0.00–0.00
	rye	509	0	0.00	0.00–0.66	0.00	0.00–0.00	0.00	0.00–0.00
	smooth brome	651	0	0.00	0.00–0.52	0.00	0.00–0.00	0.00	0.00–0.00
	oats	716	0	0.00	0.00–0.47	0.00	0.00–0.00	0.00	0.00–0.00
	triticale	1531	0	0.00	0.00–0.22	0.00	0.00–0.00	0.00	0.00–0.00
	wheat	1641	0	0.00	0.00–0.21	0.00	0.00–0.00	0.00	0.00–0.00
	tall oat-grass	1811	0	0.00	0.00–0.19	0.00	0.00–0.00	0.00	0.00–0.00
MT-8	wheat	1641	219	13.35	11.76–15.05	34.93	27.66–43.56	4.66	3.56–5.98
	triticale	1531	60	3.92	3.02–4.97	26.98	17.77–37.78	1.06	0.64–1.56
	quackgrass	3659	89	2.43	1.97–2.97	17.01	11.04–24.52	0.41	0.25–0.62
	tall oat-grass	1811	21	1.16	0.73–1.72	5.57	3.76–7.38	0.06	0.03–0.10
	smooth brome	651	7	1.08	0.46–2.07	6.29	3.14–9.29	0.07	0.02–0.13
	oats	716	5	0.70	0.25–1.49	5.40	2.00–10.80	0.04	0.00–0.09
	barley	447	3	0.67	0.17–1.73	9.67	6.00–13.00	0.06	0.00–0.15
	rye	509	3	0.59	0.15–1.52	8.33	6.00–12.00	0.05	0.00–0.11
MT-9	smooth brome	651	176	27.04	23.72–30.53	17.10	12.63–22.69	4.62	3.27–6.24
	barley	447	0	0.00	0.00–0.75	0.00	0.00–0.00	0.00	0.00–0.00
	rye	509	0	0.00	0.00–0.66	0.00	0.00–0.00	0.00	0.00–0.00
	oats	716	0	0.00	0.00–0.47	0.00	0.00–0.00	0.00	0.00–0.00
	triticale	1531	0	0.00	0.00–0.22	0.00	0.00–0.00	0.00	0.00–0.00
	wheat	1641	0	0.00	0.00–0.21	0.00	0.00–0.00	0.00	0.00–0.00
	tall oat-grass	1811	0	0.00	0.00–0.19	0.00	0.00–0.00	0.00	0.00–0.00
	quackgrass	3659	0	0.00	0.00–0.09	0.00	0.00–0.00	0.00	0.00–0.00
MT-10	smooth brome	651	22	3.38	2.17–4.95	13.05	9.36–16.95	0.44	0.24–0.68
	barley	447	0	0.00	0.00–0.75	0.00	0.00–0.00	0.00	0.00–0.00
	rye	509	0	0.00	0.00–0.66	0.00	0.00–0.00	0.00	0.00–0.00
	oats	716	0	0.00	0.00–0.47	0.00	0.00–0.00	0.00	0.00–0.00
	triticale	1531	0	0.00	0.00–0.22	0.00	0.00–0.00	0.00	0.00–0.00
	wheat	1641	0	0.00	0.00–0.21	0.00	0.00–0.00	0.00	0.00–0.00
	tall oat-grass	1811	0	0.00	0.00–0.19	0.00	0.00–0.00	0.00	0.00–0.00
	quackgrass	3659	0	0.00	0.00–0.09	0.00	0.00–0.00	0.00	0.00–0.00
MT-12	tall oat-grass	1811	6	0.33	0.13–0.67	9.67	6.83–12.67	0.03	0.01–0.06
	quackgrass	3659	6	0.16	0.07–0.33	8.67	3.17–14.33	0.01	0.00–0.03
	barley	447	0	0.00	0.00–0.75	0.00	0.00–0.00	0.00	0.00–0.00
	rye	509	0	0.00	0.00–0.66	0.00	0.00–0.00	0.00	0.00–0.00
	smooth brome	651	0	0.00	0.00–0.52	0.00	0.00–0.00	0.00	0.00–0.00
	oats	716	0	0.00	0.00–0.47	0.00	0.00–0.00	0.00	0.00–0.00
	triticale	1531	0	0.00	0.00–0.22	0.00	0.00–0.00	0.00	0.00–0.00
	wheat	1641	0	0.00	0.00–0.21	0.00	0.00–0.00	0.00	0.00–0.00
MT-13	quackgrass	3659	26	0.71	0.47–1.02	12.31	6.23–20.08	0.09	0.04–0.16
	tall oat-grass	1811	3	0.17	0.04–0.43	2.33	1.00–5.00	0.00	0.00–0.01
	barley	447	0	0.00	0.00–0.75	0.00	0.00–0.00	0.00	0.00–0.00
	rye	509	0	0.00	0.00–0.66	0.00	0.00–0.00	0.00	0.00–0.00
	smooth brome	651	0	0.00	0.00–0.52	0.00	0.00–0.00	0.00	0.00–0.00
	oats	716	0	0.00	0.00–0.47	0.00	0.00–0.00	0.00	0.00–0.00
	triticale	1531	0	0.00	0.00–0.22	0.00	0.00–0.00	0.00	0.00–0.00
	wheat	1641	0	0.00	0.00–0.21	0.00	0.00–0.00	0.00	0.00–0.00

S1 Table continued

WCM Lineage	Host plant species	n	k	P	95% CI	I	95% CI	D	95% CI
MT-14	smooth brome	651	6	0.92	0.37–1.86	17.50	9.00–25.83	0.16	0.03–0.33
	barley	447	0	0.00	0.00–0.75	0.00	0.00–0.00	0.00	0.00–0.00
	rye	509	0	0.00	0.00–0.66	0.00	0.00–0.00	0.00	0.00–0.00
	oats	716	0	0.00	0.00–0.47	0.00	0.00–0.00	0.00	0.00–0.00
	triticale	1531	0	0.00	0.00–0.22	0.00	0.00–0.00	0.00	0.00–0.00
	wheat	1641	0	0.00	0.00–0.21	0.00	0.00–0.00	0.00	0.00–0.00
	tall oat-grass	1811	0	0.00	0.00–0.19	0.00	0.00–0.00	0.00	0.00–0.00
	quackgrass	3659	0	0.00	0.00–0.09	0.00	0.00–0.00	0.00	0.00–0.00
MT-15	wheat	1641	1	0.06	0.00–0.27	13.00	0.00–0.00	0.01	0.00–0.02
	barley	447	0	0.00	0.00–0.75	0.00	0.00–0.00	0.00	0.00–0.00
	rye	509	0	0.00	0.00–0.66	0.00	0.00–0.00	0.00	0.00–0.00
	smooth brome	651	0	0.00	0.00–0.52	0.00	0.00–0.00	0.00	0.00–0.00
	oats	716	0	0.00	0.00–0.47	0.00	0.00–0.00	0.00	0.00–0.00
	triticale	1531	0	0.00	0.00–0.22	0.00	0.00–0.00	0.00	0.00–0.00
	tall oat-grass	1811	0	0.00	0.00–0.19	0.00	0.00–0.00	0.00	0.00–0.00
	quackgrass	3659	0	0.00	0.00–0.09	0.00	0.00–0.00	0.00	0.00–0.00
MT-27	wheat	1641	2	0.12	0.02–0.38	3.50	3.00–4.00	0.00	0.00–0.01
	barley	447	0	0.00	0.00–0.75	0.00	0.00–0.00	0.00	0.00–0.00
	rye	509	0	0.00	0.00–0.66	0.00	0.00–0.00	0.00	0.00–0.00
	smooth brome	651	0	0.00	0.00–0.52	0.00	0.00–0.00	0.00	0.00–0.00
	oats	716	0	0.00	0.00–0.47	0.00	0.00–0.00	0.00	0.00–0.00
	triticale	1531	0	0.00	0.00–0.22	0.00	0.00–0.00	0.00	0.00–0.00
	tall oat-grass	1811	0	0.00	0.00–0.19	0.00	0.00–0.00	0.00	0.00–0.00
	quackgrass	3659	0	0.00	0.00–0.09	0.00	0.00–0.00	0.00	0.00–0.00
MT-28	tall oat-grass	1811	1	0.06	0.00–0.24	5.00	0.00–0.00	0.00	0.00–0.01
	barley	447	0	0.00	0.00–0.75	0.00	0.00–0.00	0.00	0.00–0.00
	rye	509	0	0.00	0.00–0.66	0.00	0.00–0.00	0.00	0.00–0.00
	smooth brome	651	0	0.00	0.00–0.52	0.00	0.00–0.00	0.00	0.00–0.00
	oats	716	0	0.00	0.00–0.47	0.00	0.00–0.00	0.00	0.00–0.00
	triticale	1531	0	0.00	0.00–0.22	0.00	0.00–0.00	0.00	0.00–0.00
	wheat	1641	0	0.00	0.00–0.21	0.00	0.00–0.00	0.00	0.00–0.00
	quackgrass	3659	0	0.00	0.00–0.09	0.00	0.00–0.00	0.00	0.00–0.00
MT-29	tall oat-grass	1811	1	0.06	0.00–0.24	164.00	0.00–0.00	0.09	0.00–0.27
	barley	447	0	0.00	0.00–0.75	0.00	0.00–0.00	0.00	0.00–0.00
	rye	509	0	0.00	0.00–0.66	0.00	0.00–0.00	0.00	0.00–0.00
	smooth brome	651	0	0.00	0.00–0.52	0.00	0.00–0.00	0.00	0.00–0.00
	oats	716	0	0.00	0.00–0.47	0.00	0.00–0.00	0.00	0.00–0.00
	triticale	1531	0	0.00	0.00–0.22	0.00	0.00–0.00	0.00	0.00–0.00
	wheat	1641	0	0.00	0.00–0.21	0.00	0.00–0.00	0.00	0.00–0.00
	quackgrass	3659	0	0.00	0.00–0.09	0.00	0.00–0.00	0.00	0.00–0.00
